# Handbook of the Diagnosis of Treatment of Diseases of the Throat, Nose and Naso-pharynx

**Published:** 1883-06

**Authors:** 


**Handbook of the Diagnosis of Treatment of Diseases of the Throat, Nose and Naso-pharynx.** By CARL SEILER, M. D., Lecturer on Laryngoscopy at the University of Pennsylvania, Chief of the Throat Dispensary at the University Hospital, etc. Second edition, thoroughly revised and greatly enlarged, with seventy-seven illustrations. Philadelphia: H. C. Lea's Son & Co. 1883.

Dr. Seiler is one of the teachers in the post graduate course in the University of Pennsylvania, and the writer can testify, by personal knowledge, of his rare ability as a clinical instructor. This book demonstrates, that as an author, he possesses the same faculty of teaching, and the student of laryngoscopy will find this work an invaluable guide in acquiring the skill requisite to the successful diagnosis and treatment of diseases of the larynx and naso-pharynx. The value of the book is increased by the admirable wood-cuts with which it is illustrated.

